# Effects of captivity and artificial breeding on microbiota in feces of the red-crowned crane (*Grus japonensis*)

**DOI:** 10.1038/srep33350

**Published:** 2016-09-15

**Authors:** Yuwei Xie, Pu Xia, Hui Wang, Hongxia Yu, John P. Giesy, Yimin Zhang, Miguel A. Mora, Xiaowei Zhang

**Affiliations:** 1 State Key Laboratory of Pollution Control & Resource Reuse, School of the Environment, Nanjing University, Nanjing, China; 2 Administration of Yancheng National Natural Reserve, Yancheng, China; 3 Department of Veterinary Biomedical Sciences and Toxicology Centre, University of Saskatchewan, Saskatoon, Saskatchewan, Canada; 4 Department of Zoology, and Center for Integrative Toxicology, Michigan State University, East Lansing, MI, USA; 5 School of Biological Sciences, University of Hong Kong, Hong Kong, SAR, China; 6 Nanjing Institute of Environmental Sciences (NIES), Ministry of Environmental Protection, Nanjing, China; 7 Department of Wildlife and Fisheries Sciences, Texas A&M University, College Station, TX, USA

## Abstract

Reintroduction of the threatened red-crowned crane has been unsuccessful. Although gut microbiota correlates with host health, there is little information on gut microbiota of cranes under different conservation strategies. The study examined effects of captivity, artificial breeding and life stage on gut microbiota of red-crown cranes. The gut microbiotas of wild, captive adolescent, captive adult, artificially bred adolescent and artificially bred adult cranes were characterized by next-generation sequencing of 16S rRNA gene amplicons. The gut microbiotas were dominated by three phyla: *Firmicutes* (62.9%), *Proteobacteria* (29.9%) and *Fusobacteria* (9.6%). *Bacilli* dominated the ‘core’ community consisting of 198 operational taxonomic units (OTUs). Both captivity and artificial breeding influenced the structures and diversities microbiota of the gut. Especially, wild cranes had distinct compositions of gut microbiota from captive and artificially bred cranes. The greatest alpha diversity was found in captive cranes, while wild cranes had the least. According to the results of ordination analysis, influences of captivity and artificial breeding were greater than that of life stage. Overall, captivity and artificial breeding influenced the gut microbiota, potentially due to changes in diet, vaccination, antibiotics and living conditions. Metagenomics can serve as a supplementary non-invasive screening tool for disease control.

According to the Worldwatch Institute, populations of some avian species are currently declining worldwide, with 1,200 species facing extinction in the next century^1^. Among these species, the red-crowned crane (*Grus japonensis*) is one of the rarest cranes in the world, with approximately 2,750 mature individuals across East Asia^2^. Efforts, including establishment of biosphere reserves and artificial breeding programs, have been developed to try to maintain captive cranes for producing individuals for reintroduction into the wild^2^.

Reintroduction of both captive and artificially bred *G. japonensis* is still limited by some logistical issues, such as outbreaks of infectious diseases and limited survival of individuals raised in captivity, then reintroduced to the wild^3^,^4^,^5^,^6^. Artificial breeding (human assisted breeding) has been successful for approximately 10% of avian species overall, and artificial breeding is usually difficult for highly mobile species such as migratory cranes^1^. In addition, bacterial infections are common causes of avian disease and often contribute to overall mortality. Specifically, cranes are threatened by infectious diseases caused by bacterial pathogens such as *Clostridium perfringens*^7^, *Clostridium piliforme*^8^, *Mycobacterium spp.* and *Campylobacter*^9^,^10^,^11^. However, most assessments of health of wild individuals focus mainly on patterns of behavior^12^, psychological and physical condition^13^,^14^,^15^. Even though the gut microbiota associated with health of hosts and mortality of eggs^11^,^16^, few researches have been conducted to investigate the host-associate microbiota of *G. japonensis*.

Microbiota in the gut plays important roles in nutrition^17^,^18^, development of organs^19^,^20^,^21^ and regulation of host physiology^22^,^23^. Both captivity and artificial breeding can influence microbial communities in guts of wildlife due to vaccination against diseases, use of antibiotics to control diseases and changes in diets and living conditions^24^,^25^,^26^. However, the effects of captivity and artificial breeding on the gut microbiota of *G. japonensis* are largely unknown.

The present study was conducted to examine effects of captive breeding on gut microbiota of the threatened red-crowned cranes (*G. japonensis*). Using next-generation sequencing of 16s rRNA gene with non-invasive collection of feces, diversities of microbes in guts of cranes from three groups, wild, captive and artificially bred, were investigated. Both captive and artificially bred groupes were further divided into two life stages: adolescent and adult. Because ages of wilds crane could not be determined in the field by use of a non-invasive approach, the wild group was excluded from the part of the study that examined effects of life stages. Microbial communities in guts were assessed in five groups of *G. japonensis*, including wild, captive adolescent, captive adult, artificially bred adolescent and artificially bred adult. Artificially bred cranes were the second generation of captive adults (first generation). Specific research questions included: 1) to examine the structures of microbial communities in guts of *G. japonensis*; 2) to evaluate the effects of captivity, artificial breeding and/or host life stage on the gut microbiota of cranes.

## Results

### Next generation sequencing data

After checking qualities of sequenced reads, 2,135,253 cleaned sequences with an average length of 212 ± 13 bp (Supplementary Table S1) were obtained from 108 fecal samples. The number of sequences in each sample varied from 8,030 to 108,290 (median = 13,091). A total of 1,164 OTUs representing 25 bacterial phyla were identified in feces of *G. japonensis.* Diversities and structures of microbial communities were compared among five groups, including artificially bred adolescents (n = 23), artificially bred adults (n = 30), captive adolescents (n = 16), captive adults (n = 17) and wild cranes (n = 22) (Supplementary Table S2). Both Chao1 and phylogenetic diversity were in accordance with the observed number of OTUs (Supplementary Figure S1 and Table S1). According to rarefaction curves (Supplementary Figure S1) and Good’s coverage estimator (Supplementary Table S1), most of the abundant microbial OTUs in guts of cranes were captured by rarefaction at 8,030 reads per sample.

### Microbial communities in feces of G. japonensis

**Figure 1:**
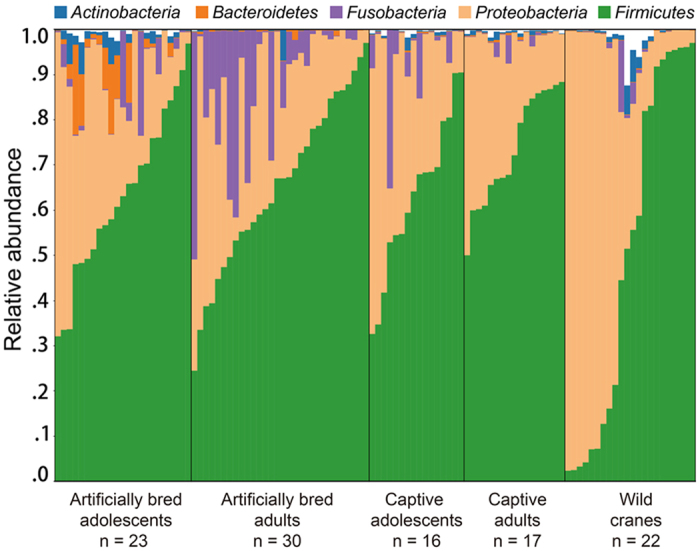
Relative abundances of the most abundant phyla in feces of red crown cranes. The 5 most abundant phyla (>1% of the total sequences) are presented. Samples are grouped according to the breeding type and age range, and arranged by the relative abundance of the most dominant phylum, *Firmicutes*.

Predominant bacterial phyla present in feces of all *G. japonensis* included *Firmicutes* (62.9 ± 4.8%), *Proteobacteria* (29.9 ± 4.7%) and *Fusobacteria* (9.6 ± 3.0%) (Fig. 1). *Clostridia* and *Bacilli* were predominant within the *Firmicutes*. Within the *Proteobacteria, Alphaproteobacteria, Epsilonproteobacteria* and *Gammaproteobacteria* were most abundant. The remaining 22 phyla with lesser abundances accounted for only 2.6% of the gut microbiota. At the genus level, the five most abundant genera were *Enterococcus* (19.1 ± 2.1%, prevalence 100%), *Bacillus* (12.2 ± 1.5%, prevalence 96.3%), *Psychrobacter* (9.3 ± 1.1%, incidence 94.4%), *Lactobacillus* (7.4 ± 1.0%, prevalence 93.5%) and *Pseudomonas* (5.4 ± 1.7%, prevalence 88.9%).

### Association network among host-associate core microbiota in feces of G. japonensis

**Figure 2:**
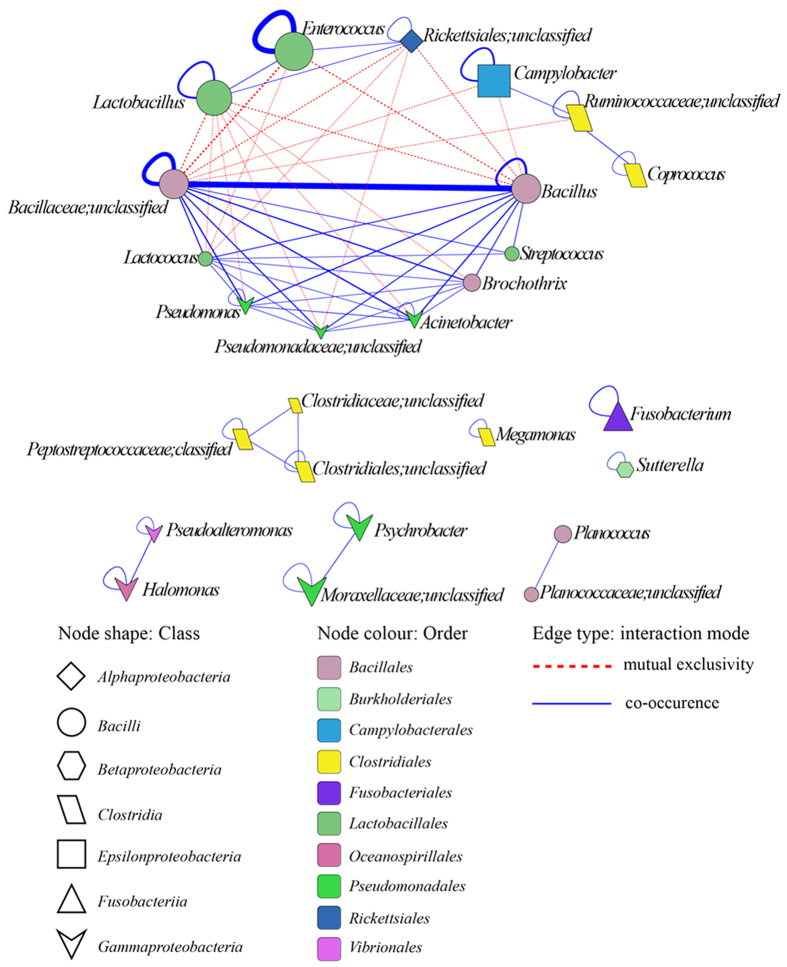
Association network of core OTUs collapsed at the genus level. Shape of node, class; color of node, order; red dashed edges, mutual exclusivity; blue edge, co-occurrence. The widths of the lines represent the relative abundances of OTUs.

There were 198 OTUs shared by more than half of all samples (n = 54). These shared OTUs of fecal microbiota composed host associated ‘core microbiota’, which were mainly within the *Gammaproteobacteria, Bacilli* and *Clostridia*. Core microbiota accounted for 86.1% of the microbiota in feces, and members of the class *Bacilli* dominated the core microbiota by accounting for 51.9% of the microbiota and 60.3% of the core microbiota. The relationship within core microbiota, co-occurrence and co-exclusion relationships among the core OTUs were inferred from the association network (Supplementary Figure S2). There were 1,135 statistically significant associations among core OTUs (‘two-tailed’ bootstrapped P value < 0.002). The topology of the network suggested that this network had a modular structure (modularity index, 0.48 > 0.4)^27^. The network comprised highly connected OTUs (≥5 edges per node, 62% of the total nodes) that can form a clustered topology. Core OTUs from the same order tended to co-occur more frequently (contributing 74.5% of the total associations) than those in different orders (contributing 25.5% of the total associations, Supplementary Figure S2). Association networks were collapsed at the genus level to identify key network hubs that modulated the gut microbiota. *Bacillus, Brochothrix, Acinetobacter, Pseudomonas, Lactococcus, Lactobacillus* and *Enterococcus* were defined as key hubs (highly connected genera, ≥5 genera, Fig. 2). *Campylobacter, Enterococcus* and *Lactobacillus* were mutually exclusive with other key hubs. Among the *Bacilli, Lactobacillus* and *Enterococcus* were mutually exclusive with *Bacillus* and *Brochothrix*.

### Microbial communities among feces of artificial bred, captive and wild groups were distinct

**Figure 3:**
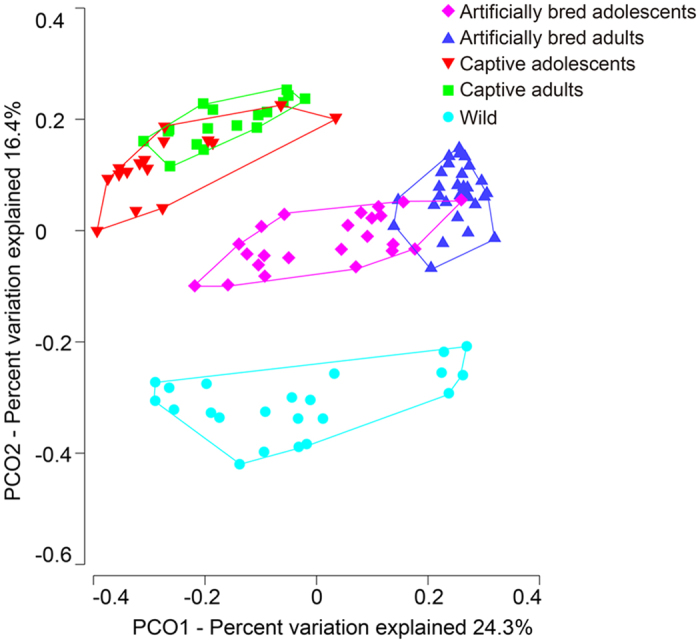
PCoA ordination of unweighted UniFrac distances revealing the influence of captivity, artificial breeding and life stage on community structuring. Distances among centroids of three groups were 0.404 (artificially bred to captive group), 0.448 (artificially bred to wild group) and 0.450 (captive to wild group). The influence of captivity and artificial breeding were greater than that of life stage (the distance between centroids of adolescent and adult cranes: 0.189).

**Figure 4:**
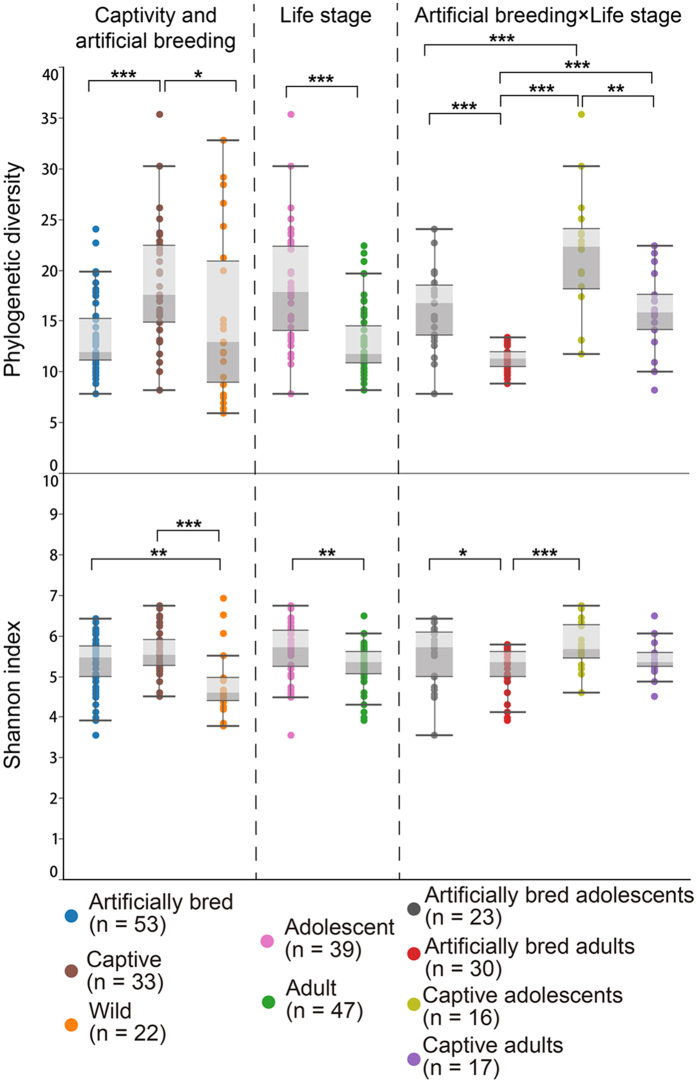
Comparisons of phylogenetic diversity and Shannon index among groups. The Kruskal-Wallis test was performed, followed by post hoc Mann-Whitney U tests. Significance was determined at P values < 0.001 (***), < 0.01 (**), and < 0.05 (*).

**Figure 5:**
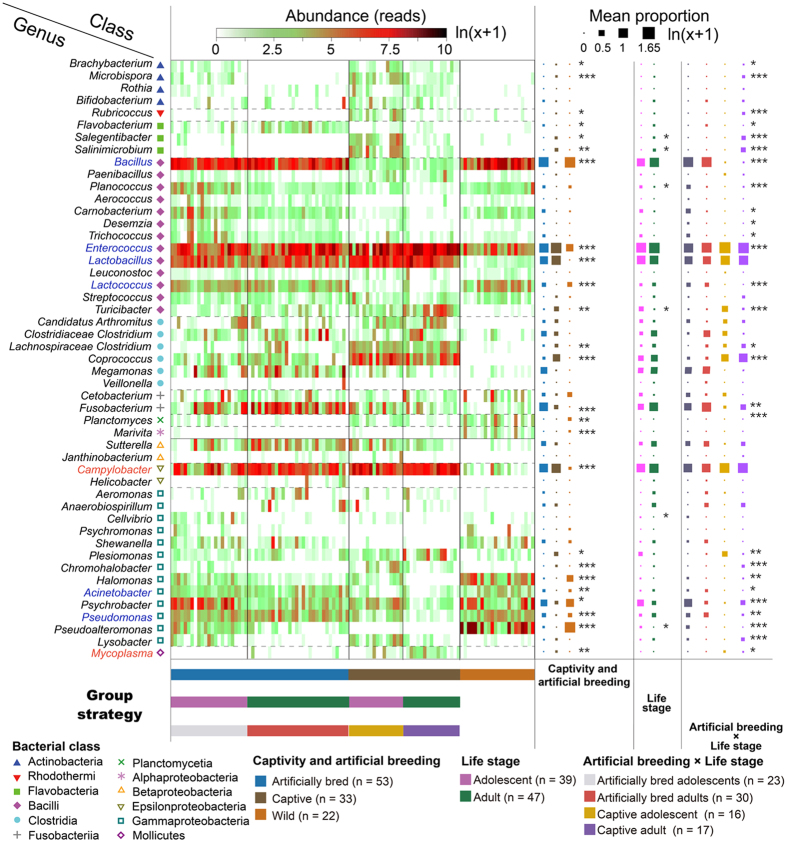
Patterns of abundance and mean relative abundances of the most abundant, assignable genera varied among groups. Criteria for abundant assignable genera are mean relative abundance > 1% and with genus annotation (annotation confidence score > 60%). Key hubs (determined by network analysis) were in blue, and *Campylobacter* (mutually exclusive with key hubs) were in red. Mean relative abundance was tested with ANOVA, P values < 0.05, followed by post hoc Tukey-Kramer tests among groups. P values were corrected using the Benjamin-Hochberg FDR method.

Both captivity and artificial breeding strongly influenced structures of fecal microbiota from three groups of *G. japonensis* (Fig. 3). Structures of fecal microbiota were also compared among artificially bred, captive and wild groups by use of the permutational multivariate analysis of variance test with unweighted UniFrac distance (PERMANOVA, Table 1). Pairwise tests revealed that compositions of microbial communities in guts of cranes from each group were each significantly different from the two other groups (Table 2). Significant differences were also observed in both Shannon index and phylogenetic diversity among groups (Kruskal-Wallis test, P < 0.05, followed by post-hoc Mann-Whitney U tests among groups, Fig. 4). The greatest alpha diversity was found in the captive group, while the wild group had the least. Patterns of classified abundant genera (max relative abundance > 1%) varied among groups (ANOVA test, P value < 0.05, followed by the post-hoc Tukey-Kramer test among groups. P values were corrected using the Benjamin-Hochberg FDR method, Fig. 5). Twenty-five genera with significantly different relative abundances among groups were identified. Most genera belonged to one of 7 classes (*Actinobacteria, Flav*o*bacteria, Bacilli, Clostridia, Gammaproteobacteria, Fusobacteria and Epsilonproteobacteria*). The relative abundances of key hubs (*Bacillus, Acinetobacter, Pseudomonas, Lactococcus, Lactobacillus* and *Enterococcus*) were significantly influenced by captivity and artificial breeding, as well as the *Campylobacter*.

### Composition of microbial communities differed among life stages in each artificially bred or captive group

Structures of microbial communities in feces displayed significant separation between life stages in both captive and artificially bred groups (Fig. 3). PERMANOVA results (Table 1) were used as an indicator of true differences and used to classify distinct groups. Compositions of microbial communities for each sub-group were significantly different from those of the three other sub-groups (Table 2, Artificial breeding **×** Life stage). Both artificially bred and captive adolescents exhibited significantly greater alpha diversities, than did adults (Fig. 4). Gut microbiota of adults contained significantly greater relative abundances of the genera *Salegentibacter* and *Salinimicrobium*. Abundances of the genera *Planococcus, Turicibacter, Cellvibrio* and *Pseudoalteromonas* were less (Fig. 5). During each life stage, artificial breeding influenced phylogenetic diversity, relative abundance of five key hubs (*Bacillus, Acinetobacter, Pseudomonas, Lactococcus* and *Enterococcus*) and structures of the gut microbiota.

### Opportunistic zoonotic bacteria in feces identified by non-invasive next-generation sequencing of 16S rRNA gene amplicon

There were 23 opportunistic enteric zoonotic pathogenic OTUs (Supplementary Table S3) representative of four classes of enteric zoonotic bacteria (Table 3) in microbiota of feces of *G. japonensis*. In this study, the V4 fragment of 16s rRNA was not sufficiently long to classify *Campylobacter spp.* and *Mycoplasma spp.* to species. Not all species within *Campylobacter* are pathogens. Incidences and relative abundances of *Campylobacter spp.* and *Clostridium piliforme* in feces of domestic cranes were significantly greater than those of wild cranes. Captive cranes harbored the greatest relative abundance of *Clostridium perfringens*. The community of microbes in feces can serve as a supplementary, non-invasive tool to screen for disease control, via 16s rRNA amplicons and next-generation sequencing.

## Discussion

A survey of bacterial communities in feces of *G. japonensis* provides evidence that captivity and artificial breeding significantly altered structures of the gut microbiota. Microbiota in guts of *G. japonensis* was dominated by *Firmicutes* (62.9%) which is consistent with results of previous studies of a range of avian species, such as gull^28^, Canada goose^29^, procellariform seabirds^30^ and kakapo^31^,^32^, where members of the phylum *Firmicutes* dominated microbiota of guts. *Firmicutes* are associated with breakdown of complex carbohydrates and fatty acids, which produce energy for the host^33^,^34^. Relative abundances of *Proteobacteria* (29.9%) *and Fusobacteria* (9.6%) in *G. japonensis* were greater than those in gull, Canada goose, or kakapo, while relative abundances of *Bacteroidetes* (1%) *and Actinobacteria* (0.9%) were less^16^,^25^,^28^,^29^,^30^. *Gammaproteobacteria* were present in feces of all *G. japonensis* as well as in feces of kakapo, turkey and the Adelie penguin (*Pygoscelis adeliae*)^23^,^32^. Similar to microbiota in guts of *Pachyptila turtur* and *Pelecanoides urinatrix*, microbiota in guts of *G. japonensis* contained *Fusobacteria*, potentially due to their diets high in fat^30^,^35^. In general, the top two most abundant phyla in gut microbiota of omnivorous red-crown crane were similar to that of omnivorous gull and herbivores Canada goose and kakapo. However, variations of relative abundances of the dominated phyla among avian species may be driven by the host genetics and diets^23^.

The association network of host associated core microbiota suggested that succession pattern of microbial communities in *G. japonensis* gut were mainly driven by interactions among *Lactobacillus, Enterococcus, Campylobacter*, and *Bacillus. Lactobacillus* and *Enterococcus* were co-occurred, which might be due to nutritional cross-feeding and co-colonization^36^,^37^. Generally, most of the co-occurring species pairs or assemblages might share similar ecological characteristics^38^,^39^. On the other hand, *Lactobacillus* and *Enterococcus* were mutually exclusive with *Bacillus*, which might be due to toxic products, habitation modification, the detriment of microbial neighbors and immunomodulation^36^,^37^. Furthermore, *Campylobacter,* a potential pathogen, was mutually exclusive with *Bacillus*, while there were no significant associations of *Campylobacter* with any other taxonomic group in poultry-associated microbial network^40^.

Being held in captivity significantly influenced the assemblages of microbiota in guts of *G. japonensis, which* is consistent with results of previous studies on other animals, including endangered Kakapo, Antarctic seals and wild-caught rodents^31^,^41^,^42^. Specifically, domestic *G. japonensis* might not develop a wild-type gut microbiota during rearing in captivity. The alpha-diversity, structures of gut microbiota of captive cranes and relative abundances of key hubs were significantly different from those of wild cranes. Among the three groups, captive cranes exhibited the greatest diversity their gut microbiota, while wild cranes had the least. This might be related to transmission of bacteria, such as constant social interactions with human keepers^41^. Wild-caught animals might retain hallmarks of their previous gut microbiota for extended periods^42^,^43^. However, according to the shorter distance between centroids of captive and artificially bred groups than others (Fig. 3), since both captive and artificially bred cranes received a uniform diet and medical care in the reserve, captivity might have greater effects on gut microbiota than retained previous gut microbial communities of captive cranes^24^,^25^. Furthermore, changes in gut microbiota of domestic cranes might also be caused by exposure to antibiotics. Domestic cranes are fed fish that are obtained from aquaculture facilities, which routinely use antibiotics to control disease in fish held at high population densities during aquaculture^44^. Domestic cranes are also routinely, prophylactically administered antibiotics to protect them from diseases often encountered while in captivity. Exposure to antibiotics might cause long-term changes in relative abundances of species within the microbial community of the gut. These sorts of changes have been observed in gut microbiota of humans treated with antibiotics^45^. Among the significantly different genera between captive and wild groups, *Lactobacillus* (class *Bacilli*) was the taxa with the highest fold change (461-fold greater in captive group than in wild group, Fig. 5). *Lactobacillus* were represented in gut microbiota community that exhibited elevated β-xylosidase and β-glucosidase levels^23^. The greater relative abundance of *Lactobacillus* in both captive and artificially bred groups than in the wild group might be due to the formula diet which contained relatively large amounts of sugar.

Besides captivity, artificial breeding also exerted a strong influence on microbiota in guts of *G. japonensis*. Microbial communities in guts of artificially bred adults were distinct from those of captive adults who had hatched in the wild but were raised under the same conditions as the artificially bred adults. Artificially bred cranes lost chances of interactions with their parents, compared with other naturally hatched cranes. Artificially bred cranes were caged together since they had been hatched. Compared with wild cranes, relatively great rearing density, formula diet and frequent contact with human keepers of artificially bred cranes might increase risk of infection from the time they were neonates. The greater relative abundance of butyrate-producing *Fusobacteria* in artificially bred cranes might act as a feedback to defend against potentially pathogenic bacteria^30^,^46^, which revealed that risks of infection for cranes in the ambient environment might be greater than in the wild.

Success of reintroduction of captive birds into the wild may depend on development of microbial assemblies in the gut^26^. Separation of structures of microbial communities in feces between the adolescent and adult cranes suggests *G. japonensis* gut microbiota transition following the change of life stage. Adolescent *G. japonensis* exhibited significantly greater alpha diversities than did adults. Changes of structures and alpha-diversities of gut microbiota during host development have been reported in fishes^19^, mammals^47^,^48^ and kakapos^31^. Adolescent animals were mainly influenced by exposure to bacteria in their environment during early development^19^, especially for development of the immature immune system^49^. One possible way to manage this development would be to treat individuals with probiotics and/or “microbe” transplants to maintain relatively stable gut microbiota^50^,^51^.

In the present study, we delineate the diversities and structures of fecal microbiota of wild, captive and artificially bred red-crown cranes. The gut microbiota of *G. japonensis* in captivity and artificial breeding groups were significantly altered from the wild group, which might increase the risk of infection and further cause the failure of reintroduction. Building upon the current observations, further studies may combine multiple “omics” technologies to identify the effects of diet change and use of antibiotics on the health of cranes. Fecal microbiota analysis could be a useful tool to assess the health status of hosts and to improve conservation and management strategies for this threatened species, if the references of gut microbiota of healthy cranes were characterized.

## Methods

### Ethics statement

All methods, procedures and, experiments were conducted in accordance with the guidelines issued by the Ethics Committee of Nanjing University. All experimental designs and methods for sampling have been approved by the Administration of the Yancheng National Natural Reserve in China.

### Sample description

Samples of feces were collected from both domestic and wild *G. japonensis* at the Yancheng National Nature Reserve (32.614~34.476N, 119.857~121.096E) on January 10, 2013. Cranes in captivity received a uniform diet and medical care in the reserve. Also, based on regulations for breeding programs for birds, individual cranes had the same amount of interaction with the same keepers. The captive cranes (17 individuals) and the artificially bred cranes (28 individuals) were kept separated in different enclosures according to age. Artificially bred cranes were the second generation of captive adult cranes (first generation). Each bonded, pair of adults used for artificial breeding (>3 years old), was housed in a single enclosure before and during breeding. Artificially bred adolescents (<3 years) were reared together in two separate enclosures (one for individuals of about 1-year or age and the other for individuals of approximately 2-years of age. Cranes were classified as adolescents (<3 years, sexually immature) or adults (>3 years, sexually mature) based on morphology of both feathers and topknot of adults^52^. Feces of wild *G. japonensis* were collected from 11 individuals at an area in which they foraged. Samples of feces were placed into sterile 5-mL storage tubes and transferred on ice to the laboratory. A total of 108 samples of feces were collected (Supplementary Table S2). Samples were vacuum freeze-dried, homogenized and stored at −80 °C prior to extraction of DNA.

### Extraction of DNA, amplification by PCR and next-generation sequencing

Extraction of DNA from a 0.20 g portion of each fecal sample was performed using the MoBio Power Soil DNA Kit (Mo Bio Laboratories Inc., CA, USA) following the manufacturer’s protocol. Extracted DNA was quantified and evaluated for purity prior to storage at −80 °C. The hypervariable V4 region of the bacterial 16S rRNA gene was amplified using the 563F (5′-AYTGGGYDTAAAGNG-3′) and V4R primers (an equal mixture of four primers: 5′-TACCRGGGTHTCTAATCC-3′, 5′-TACCAGAGTATCTAATTC-3′, 5′-CTACDSRGGTMTCTAATC-3′, and 5′-TACNVGGGTATCTAATCC-3′) (RDP’s Pyrosequencing Pipeline: http://pyro.cme.msu.edu/pyro/help.jsp). A 20-μL reaction including Platinum^®^ Taq polymerase (Life Technologies, CA, USA) was used for each PCR amplification. Amplification was conducted in a SureCycler 8800 Thermal Cycler (Agilent Technologies, CA, USA) under the following conditions: initial denaturation at 94 °C for 2 min followed by 25 cycles at 94 °C for 15 s, 50 °C for 30 s and 68 °C for 30 s, with a final extension at 68 °C for 7 min. To minimize potential bias during PCR triplicate reactions were performed for each sample. Products of PCR were checked to assess their size and specificity by use of electrophoresis on 1.2% w/v agarose gels, and purification was performed using the MinElute Gel Extraction Kit (Qiagen, CA, USA). Purified products were quantified using Qubit™ dsDNA HS Assay Kits (Invitrogen, CA, USA) and then adjusted to 10 ng/μL with molecular-grade water. All purified products were pooled at an equal ratio for subsequent sequencing using an Ion Torrent Personal Genome Machine (PGM, Life Technologies, CA, USA) according to the manufacturer’s instructions.

### Bioinformatics and statistical analysis

Sequenced amplicons were analyzed following the UPARSE pipeline^53^. *Quality control*: PGM adaptor sequences were automatically removed on the ION server (version 3.6.2). Primer sequences and low-quality reads were discarded from the raw output by use of the QIIME toolkit^54^ (*split_libraries.py* script: average quality cut-off value: 20; sliding window size: 50 bp; maximum length of homopolymer: 8; minimum sequence length: 200 bp; maximum sequence length: 260 bp). *Denoising, chimera removal and OTU-picking*: Cleaned reads were denoised and clustered into OTUs with a sequence similarity cut-off of 97%. Chimeric and singleton OTUs (sizes less than 2) were removed. After evaluation of the quality of the sequencing and application of a chimeric reads filter, a total of 2,135,253 clean sequences with an average length of 212 ± 13 bp were obtained (Supplementary Tables S1). *Taxonomic assignment and cleaning*: Taxonomy was assigned to representative sequences using the RDP classifier^55^ against the Greengenes database (bacterial OTUs)^56^. A small number of unexpected archaeal and chloroplast sequences were removed (QIIME *filter_taxa_from_otu_table.py* script: -p k__Bacteria -n c__Chloroplast). *Alpha diversity*: Alpha rarefaction was performed using the Shannon, Phylogenetic Diversity (PD), Chao1 and observed species metrics (Supplementary Table S1). *Beta diversity and clustering*: All samples were rarefied at an equal sequencing depth (8,030 reads/sample) to reduce biases resulting from differences in sequencing depth. Principal coordinate analysis (PCoA) was performed to cluster the microbial communities based on the unweighted UniFrac distance.

Comparisons of phylogenetic diversity and evenness were performed, using the Kruskal-Wallis test, followed by post hoc Mann-Whitney U tests in the R environment. Pairwise comparisons of abundant OTUs (relative abundance > 1%) were performed using ANOVA followed by post-hoc Tukey-Kramer tests. P values were corrected using Benjamin-Hochberg FDR methods. PCoA and PERMANOVA were used to analyze the bacterial community data with PRIMER 7 software^57^,^58^. The statistical significance was set as P value < 0.05, and the number of permutation test replicates was set at 9,999. An association network of core OTUs was generated using SparCC^59^ with 500 bootstraps to assign P values. Core OTUs were OTUs that were found in more than 50% of all samples. The microbial “core” association network was filtered to include only correlations with a correlation ρ > 0.7 and a ‘two-tailed’ P value < 0.002. The network was displayed and analyzed with Cytoscape^60^. To identify key genera that modulated the gut microbiota, the network was collapsed at the level of genus with ThematicMap (http://apps.cytoscape.org/apps/thematicmap#cy-app-releases-tab). Some topological parameters were calculated using the Network Analyzer^61^ to describe the complex pattern of interrelationships among nodes.

## Additional Information

**How to cite this article**: Xie, Y. *et al*. Effects of captivity and artificial breeding on microbiota in feces of the red-crowned crane (*Grus japonensis*). *Sci. Rep.* **6**, 33350; doi: 10.1038/srep33350 (2016).

## Supplementary Material

Supplementary Information

## Figures and Tables

**Table 1:** Effects of captivity, artificial breeding and life stage on gut microbiota assessed by PERMANOVA with unweighted UniFrac distance.

Source of variation	Dataset (cranes)	d.f.	SS	F
Captivity and artificial breeding	Artificially bred, captive and wild	2	6.20	22.91*
Captivity	Captive and wild	1	3.33	22.48*
Artificial breeding	Artificially bred and captive	1	1.35	9.23*
Life stage	Artificially bred	1	1.22	12.14*
	Captive	1	0.52	4.72*
Artificial breeding × Life stage	Artificially bred adolescents, artificially bred adults, captive adolescents, and captive adults	3	5.09	16.33*

PERMANOVA of community composition to generate a permutated F statistic (F) with calculated degrees of freedom (d.f.) and sums of squares (SS).

*Permutated P value = 0.0001, 9,999 permutations.

**Table 2:** Pair-wise, post hoc comparisons between Captivity, artificial breeding and artificial breeding × life stage using the t-statistic.

Source of variation: captivity and artificial breeding				
Dataset (group)	Wild	Artificially bred	Captive	
Wild	—			
Artificially bred	(4.33, *)	—		
Captive	(4.74, *)	(5.24, *)	—	
**Source of variation: artificial breeding × life stage**				
**Dataset (group)**	**Artificially bred**		**Captive**	
	**Adolescent**	**Adult**	**Adolescent**	**Adult**
Artificially bred adolescent	—			
Artificially bred adult	(3.48, *)	—		
Captive adolescent	(3.25, *)	(5.75, *)	—	
Captive adult	(3.32, *)	(5.32, *)	(2.17, *)	—

t value and statistical significance are in parentheses. *P value ≤ 0.001.

**Table 3:** Incidences, means and ranges of relative abundance of opportunistic enteric zoonotic pathogens in feces of cranes.

Pathogen	Prevalence, mean and range of relative abundance (%)				
	Artificially bred		Captive		Wild
	Adolescent	Adult	Adolescent	Adult	
*Campylobacter spp.*	100	96.97	100	100	54.55
	9.52*	8.44*	15.97***	16.32***	0.25
	0.02~39.96	0~27.73	0.21~48.19	2.25~41.5	0~4.01
*Mycoplasma spp.*		16.67	50	82.35	4.55
	0	0.02	0.11	0.19**	0.0006
		0~0.36	0~0.88	0~1.28	0~0.01
*Clostridium perfringens*	21.7	43.3	93.7	94.1	9.1
	0.02	0.3	0.67	0.61	0.003
	0~0.21	0~4.92	0~1.89	0~2.54	0~0.06
*Clostridium piliforme*	30.43	40	12.5	29.41	4.55
	0.01*	0.31*	0.01*	0.07*	0.001
	0~0.15	0~4.4	0~0.07	0~0.83	0~0.02

Mean relative abundance was tested with ANOVA, followed by post hoc, Tukey-Kramer tests among groups. P values were corrected using the Benjamin-Hochberg FDR method. Mean relative abundance that significantly differed from wild group is in **bold** (*P_FDR_ < 0.05, **P_FDR_ < 0.01, ***P_FDR_ < 0.001).
