# Supplementary material for: Effects of captivity and artificial breeding on microbiota in feces of the red-crowned crane (Grus japonensis)
Source: Sci Rep. 2016 Sep 15;6:33350. doi: 10.1038/srep33350 (PMC5024133; doi:10.1038/srep33350)
Supplement: Supplementary Information [file srep33350-s1.doc]

**Effects of captivity and artificial breeding on microbiota in feces of the** **red-crowned crane (*****Grus japonensis*)**

Yuwei Xie^a^, Pu Xia^a^, Hui Wang^b^, Hongxia Yu^a^, John P. Giesy^a,c,d,e^, Yimin Zhang^g^, Miguel A. Mora^f^, Xiaowei Zhang^a*^

^a^ State Key Laboratory of Pollution Control & Resource Reuse, School of the Environment, Nanjing University, Nanjing, China

^b^ Administration of Yancheng National Natural Reserve, Yancheng, China

^c^ Department of Veterinary Biomedical Sciences and Toxicology Centre, University of Saskatchewan, Saskatoon, Saskatchewan, Canada.

^d^ Department of Zoology, and Center for Integrative Toxicology, Michigan State University, East Lansing, MI, USA

^e^ School of Biological Sciences, University of Hong Kong, Hong Kong, SAR, China

^f^ Department of Wildlife and Fisheries Sciences, Texas A&M University, College Station, TX, USA

^g^ Nanjing Institute of Environmental Sciences (NIES), Ministry of Environmental Protection, Nanjing, China

**Correspondence:** Xiaowei Zhang, School of the Environment, Nanjing University, Nanjing, 210089, China; Tel.: 86-25-83593649; Fax: 86-25-83707304; E-mail: [howard50003250@yahoo.com](mailto:howard50003250@yahoo.com)

**Supporting Information**

**FIGURE LEGENDS**

**Figure S1.** **Rarefaction curves of Shannon index, phylogenetic diversity, Chao1 index and observed number of OTUs.**

**Figure S2. Association network of core OTUs, related to Figure 2. Core OTUs that were present in more than half of samples of feces were selected.** Shape of node, class; color of node, order; label of node, family; red dashed edges, mutual exclusivity; blue edges, co-occurrence. The average network distance between all pairs of nodes (average path length) was 2.54 edges. The network diameter (longest distance) was 7 edges. The average clustering coefficient (which is a measure of the tendency to cluster together) was 0.73.

**
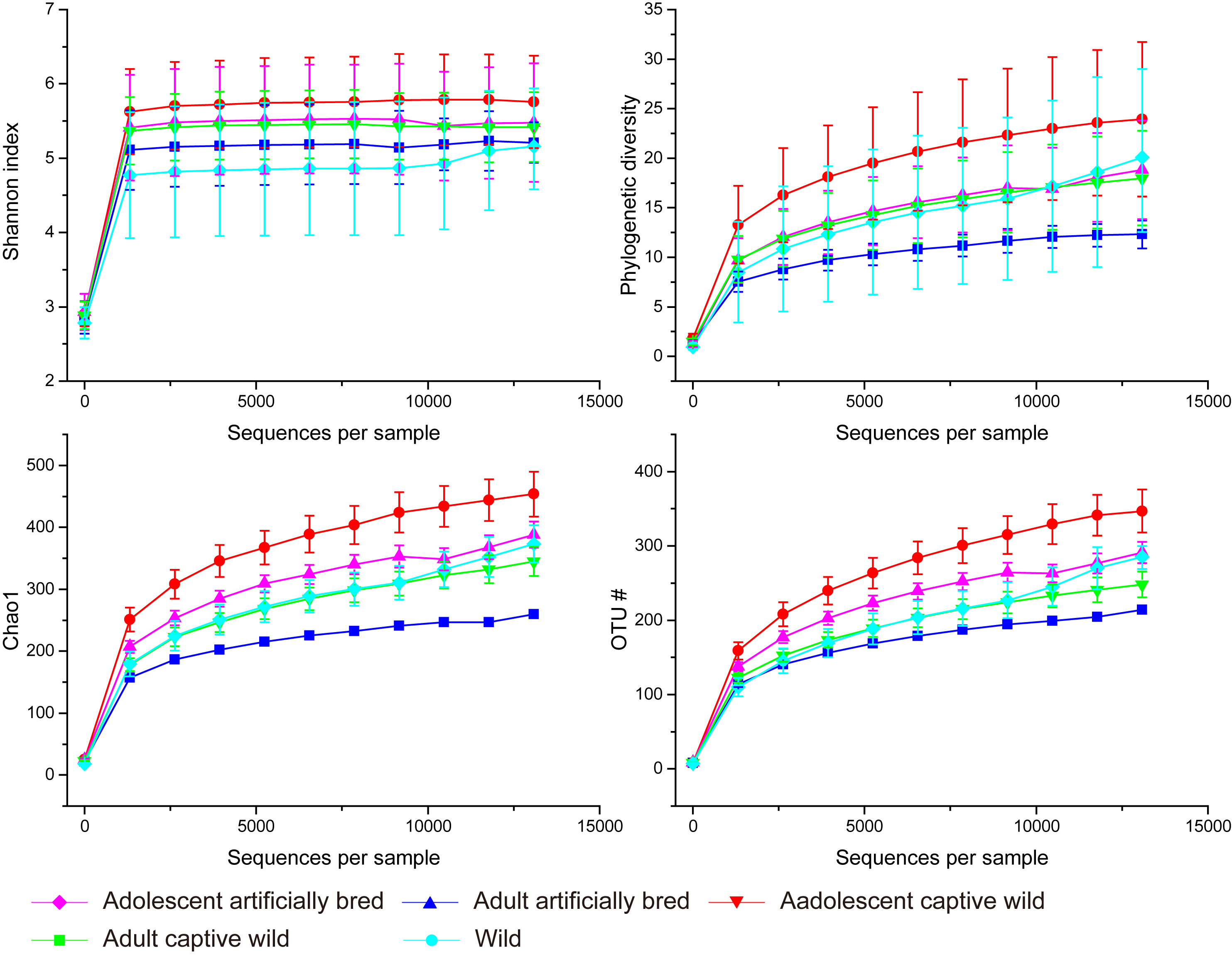
**

**Figure S1.**

**
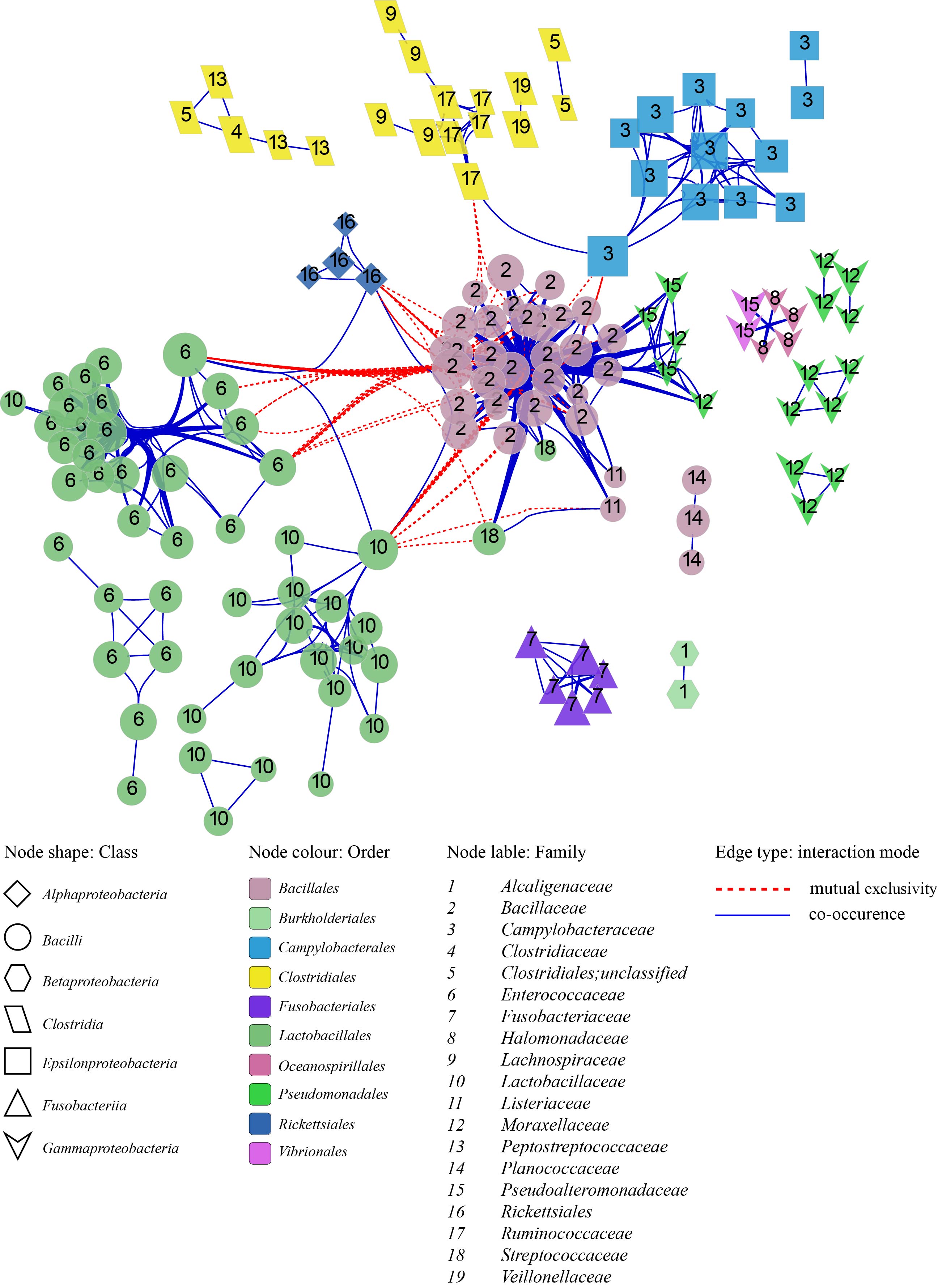
**

**Figure S2.**

**Table S1. Summary of sequencing depth and alpha diversities for each sample. Alpha diversities were calculated at an equal sequencing depth (8,030 reads / sample). OTUs # = observed number of OTUs; PD = phylogenetic diversity; Goods = Good's coverage estimator.**

| Group | ID | Reads | OTUs # | Chao1 | Shannon | PD | Goods |
| --- | --- | --- | --- | --- | --- | --- | --- |
| Artificially | 1 | 20,655 | 186 | 244.8 | 5.18 | 9.76 | 0.994 |
| bred | 2 | 15,968 | 175 | 247.1 | 4.84 | 10.86 | 0.994 |
| adults | 3 | 16,799 | 171 | 205.7 | 5.06 | 10.19 | 0.995 |
|  | 4 | 12,440 | 192 | 265.8 | 5.36 | 10.31 | 0.993 |
|  | 5 | 11,205 | 205 | 263 | 5.38 | 13.03 | 0.994 |
|  | 6 | 9,910 | 224 | 268.2 | 5.69 | 12.96 | 0.994 |
|  | 7 | 12,510 | 204 | 228 | 5.62 | 12.79 | 0.996 |
|  | 8 | 14,707 | 203 | 234.9 | 5.52 | 11.88 | 0.995 |
|  | 9 | 10,310 | 151 | 194.2 | 3.96 | 9.26 | 0.995 |
|  | 10 | 18,145 | 205 | 256.5 | 5.48 | 12.75 | 0.994 |
|  | 11 | 17,621 | 200 | 237.6 | 5.04 | 10.69 | 0.995 |
|  | 12 | 8,030 | 186 | 229 | 5.36 | 10.37 | 0.994 |
|  | 13 | 8,917 | 196 | 237 | 5.48 | 10.8 | 0.995 |
|  | 14 | 12,418 | 190 | 230 | 5.34 | 11.84 | 0.994 |
|  | 15 | 12,667 | 203 | 232 | 5.68 | 11.96 | 0.995 |
|  | 16 | 8,469 | 200 | 248.2 | 5.73 | 11.37 | 0.995 |
|  | 17 | 9,647 | 183 | 224 | 4.13 | 9.64 | 0.995 |
|  | 18 | 9,808 | 214 | 249.7 | 5.7 | 12.31 | 0.995 |
|  | 19 | 9,104 | 199 | 228 | 5.63 | 11.66 | 0.995 |
|  | 20 | 8,976 | 163 | 226.1 | 4.6 | 10.21 | 0.994 |
|  | 21 | 10,703 | 198 | 241.2 | 5.37 | 11.37 | 0.994 |
|  | 22 | 10,919 | 170 | 215.9 | 5.01 | 9.82 | 0.995 |
|  | 23 | 9,123 | 183 | 218.4 | 4.85 | 11.02 | 0.994 |
|  | 24 | 9,239 | 200 | 241 | 5.77 | 11.68 | 0.995 |
|  | 25 | 8,528 | 187 | 253 | 5.73 | 11.62 | 0.994 |
|  | 26 | 11,632 | 207 | 265.8 | 5.36 | 11.84 | 0.994 |
|  | 27 | 10,598 | 182 | 230.8 | 5.61 | 10.96 | 0.995 |
|  | 28 | 9,986 | 168 | 199.1 | 4.98 | 11.76 | 0.995 |
|  | 29 | 11,693 | 172 | 237.1 | 3.87 | 12.87 | 0.992 |
|  | 30 | 12,912 | 137 | 198.9 | 4.33 | 10.1 | 0.994 |
| Captive | 1 | 13,083 | 332 | 431.2 | 6.22 | 24 | 0.988 |
| adolescents | 2 | 18,031 | 333 | 413.9 | 5.9 | 22.79 | 0.988 |
|  | 3 | 9,061 | 297 | 403 | 5.57 | 22.84 | 0.988 |
|  | 4 | 15,086 | 292 | 432.9 | 5.51 | 20.54 | 0.985 |
|  | 5 | 13,118 | 173 | 219.9 | 5.74 | 11.74 | 0.995 |
|  | 6 | 10,250 | 375 | 488.4 | 6.44 | 26.35 | 0.987 |
|  | 7 | 10,168 | 209 | 290.4 | 5.05 | 18.16 | 0.991 |
|  | 8 | 43,485 | 327 | 412.9 | 5.18 | 24.03 | 0.987 |
|  | 9 | 35,733 | 273 | 369.8 | 5.26 | 19.38 | 0.99 |
|  | 10 | 59,086 | 159 | 219.3 | 4.56 | 12.65 | 0.994 |
|  | 11 | 29,830 | 191 | 242.1 | 5.65 | 13.26 | 0.994 |
|  | 12 | 93,073 | 239 | 279.2 | 5.53 | 17.3 | 0.994 |
|  | 13 | 67,936 | 308 | 412.1 | 5.82 | 23.42 | 0.988 |
|  | 14 | 53,656 | 431 | 583.9 | 6.68 | 30.07 | 0.984 |
|  | 15 | 108,290 | 346 | 445.7 | 6.32 | 24.01 | 0.989 |
|  | 16 | 91,897 | 510 | 680.7 | 6.67 | 34.94 | 0.981 |
| Artificially | 1 | 13,195 | 272 | 376.1 | 5.49 | 18.57 | 0.988 |
| bred | 2 | 18,966 | 366 | 550 | 6.22 | 24.53 | 0.984 |
| adolescents | 3 | 19,858 | 267 | 381.4 | 5.58 | 19.72 | 0.989 |
|  | 4 | 17,225 | 274 | 372.8 | 5.77 | 19.86 | 0.989 |
|  | 5 | 15,254 | 282 | 435.1 | 6.19 | 18.32 | 0.989 |
|  | 6 | 8,530 | 214 | 330.2 | 4.74 | 15.51 | 0.991 |
|  | 7 | 14,035 | 196 | 236 | 5.02 | 12.16 | 0.994 |
|  | 8 | 14,066 | 122 | 171.8 | 3.56 | 8.11 | 0.995 |
|  | 9 | 11,370 | 301 | 382 | 6.12 | 19.05 | 0.99 |
|  | 10 | 14,620 | 215 | 282 | 4.67 | 13.08 | 0.992 |
|  | 11 | 8,808 | 257 | 299.8 | 6.05 | 15.16 | 0.993 |
|  | 12 | 10,179 | 372 | 487 | 6.31 | 23.81 | 0.986 |
|  | 13 | 15,228 | 261 | 377.9 | 5.81 | 16.35 | 0.989 |
|  | 14 | 11,210 | 214 | 277.4 | 4.55 | 11.75 | 0.993 |
|  | 15 | 11,706 | 223 | 278.1 | 5.73 | 13.2 | 0.993 |
|  | 16 | 8,154 | 292 | 348.7 | 6.04 | 18.29 | 0.992 |
|  | 17 | 11,440 | 189 | 239.3 | 4.49 | 11.52 | 0.993 |
|  | 18 | 18,546 | 280 | 375.1 | 5.69 | 19.15 | 0.988 |
|  | 19 | 9,409 | 324 | 446.6 | 6.41 | 20.43 | 0.987 |
|  | 20 | 12,155 | 190 | 241.8 | 5 | 13.82 | 0.994 |
|  | 21 | 10,849 | 236 | 319.8 | 5.8 | 14.28 | 0.991 |
|  | 22 | 14,338 | 250 | 303.8 | 6.16 | 16.47 | 0.992 |
|  | 23 | 12,801 | 248 | 295.5 | 5.88 | 16.15 | 0.993 |
| Captive | 1 | 13,259 | 291 | 440.6 | 5.99 | 19.28 | 0.989 |
| adults | 2 | 22,435 | 346 | 422.7 | 6.5 | 22.87 | 0.988 |
|  | 3 | 92,382 | 123 | 150 | 4.52 | 8.53 | 0.997 |
|  | 4 | 13,705 | 169 | 241.5 | 4.86 | 12.5 | 0.993 |
|  | 5 | 57,654 | 196 | 296.6 | 5.35 | 15.68 | 0.993 |
|  | 6 | 50,161 | 180 | 217.4 | 5.39 | 14.48 | 0.995 |
|  | 7 | 47,218 | 266 | 366.7 | 5.62 | 21.4 | 0.989 |
|  | 8 | 14,928 | 230 | 361.4 | 5.62 | 17.6 | 0.991 |
|  | 9 | 18,021 | 281 | 404.8 | 5.29 | 21.74 | 0.988 |
|  | 10 | 11,676 | 229 | 343.5 | 5.26 | 17.28 | 0.99 |
|  | 11 | 15,268 | 225 | 391.4 | 5.17 | 16.22 | 0.989 |
|  | 12 | 11,270 | 204 | 244.3 | 5.82 | 15.18 | 0.994 |
|  | 13 | 8,646 | 232 | 310.8 | 6.05 | 16.14 | 0.992 |
|  | 14 | 13,107 | 232 | 347.7 | 5.54 | 18.17 | 0.99 |
|  | 15 | 14,018 | 211 | 296 | 5.42 | 15.4 | 0.992 |
|  | 16 | 27,855 | 144 | 205.5 | 5.24 | 11.79 | 0.995 |
|  | 17 | 28,234 | 140 | 171.2 | 5.19 | 9.77 | 0.996 |
| Wild | 1 | 16,456 | 117 | 176.1 | 3.83 | 9.6 | 0.995 |
|  | 2 | 14,941 | 393 | 502.1 | 6.51 | 27.87 | 0.987 |
|  | 3 | 10,965 | 148 | 220.5 | 3.85 | 11.62 | 0.993 |
|  | 4 | 9,586 | 180 | 245.1 | 4.38 | 13.53 | 0.992 |
|  | 5 | 10,984 | 119 | 192.5 | 3.76 | 9.02 | 0.994 |
|  | 6 | 10,469 | 122 | 169.6 | 4.51 | 6.78 | 0.995 |
|  | 7 | 20,718 | 149 | 232.2 | 4.25 | 11.29 | 0.992 |
|  | 8 | 22,353 | 362 | 479.2 | 5.58 | 25.86 | 0.985 |
|  | 9 | 12,333 | 115 | 140.8 | 4.39 | 7.27 | 0.996 |
|  | 10 | 23,209 | 333 | 434.2 | 6.01 | 23.52 | 0.987 |
|  | 11 | 18,627 | 476 | 561.6 | 6.92 | 33.33 | 0.986 |
|  | 12 | 25,293 | 275 | 376.9 | 4.98 | 19.63 | 0.988 |
|  | 13 | 24,833 | 172 | 254.5 | 4.4 | 13.94 | 0.992 |
|  | 14 | 11,932 | 108 | 153.8 | 4.19 | 6.57 | 0.996 |
|  | 15 | 10,293 | 194 | 286.9 | 4.58 | 14.38 | 0.991 |
|  | 16 | 12,813 | 118 | 149.2 | 4.48 | 6.39 | 0.996 |
|  | 17 | 23,842 | 394 | 474 | 6.52 | 29.1 | 0.988 |
|  | 18 | 20,043 | 138 | 175.3 | 4.7 | 7.73 | 0.995 |
|  | 19 | 12,789 | 288 | 395.2 | 4.92 | 20.18 | 0.987 |
|  | 20 | 11,128 | 168 | 272 | 4.57 | 12.46 | 0.992 |
|  | 21 | 13,100 | 176 | 282.5 | 4.63 | 13.06 | 0.991 |
|  | 22 | 11,345 | 227 | 308.4 | 4.91 | 15.48 | 0.991 |

**Table S2. Summary information for samples of feces, including group, age and rearing facility.**

| Group | ID | Age | Rearing facility | |
| --- | --- | --- | --- | --- |
|  |  |  | ID | NO. of cranes |
| Artificially bred adults | 1 | > 3 yrs | BF01 | 2 |
|  | 2 | > 3 yrs | BF01 | 2 |
|  | 3 | > 3 yrs | BF01 | 2 |
|  | 4 | > 3 yrs | BF01 | 2 |
|  | 5 | > 3 yrs | BF02 | 2 |
|  | 6 | > 3 yrs | BF02 | 2 |
|  | 7 | > 3 yrs | BF02 | 2 |
|  | 8 | > 3 yrs | BF02 | 2 |
|  | 9 | > 3 yrs | BF03 | 2 |
|  | 10 | > 3 yrs | BF03 | 2 |
|  | 11 | > 3 yrs | BF03 | 2 |
|  | 12 | > 3 yrs | BF03 | 2 |
|  | 13 | > 3 yrs | BF04 | 2 |
|  | 14 | > 3 yrs | BF04 | 2 |
|  | 15 | > 3 yrs | BF04 | 2 |
|  | 16 | > 3 yrs | BF04 | 2 |
|  | 17 | > 3 yrs | BF05 | 2 |
|  | 18 | > 3 yrs | BF05 | 2 |
|  | 19 | > 3 yrs | BF05 | 2 |
|  | 20 | > 3 yrs | BF05 | 2 |
|  | 21 | > 3 yrs | BF06 | 2 |
|  | 22 | > 3 yrs | BF06 | 2 |
|  | 23 | > 3 yrs | BF06 | 2 |
|  | 24 | > 3 yrs | BF06 | 2 |
|  | 25 | > 3 yrs | BF07 | 2 |
|  | 26 | > 3 yrs | BF07 | 2 |
|  | 27 | > 3 yrs | BF07 | 2 |
|  | 28 | > 3 yrs | BF08 | 2 |
|  | 29 | > 3 yrs | BF08 | 2 |
|  | 30 | > 3 yrs | BF08 | 2 |
| Artificially bred adolescents | 1 | > 90 days, < 1 yrs | R12 | 7 |
|  | 2 | > 90 days, < 1 yrs | R12 | 7 |
|  | 3 | > 90 days, < 1 yrs | R12 | 7 |
|  | 4 | > 90 days, < 1 yrs | R12 | 7 |
|  | 5 | > 90 days, < 1 yrs | R12 | 7 |
|  | 6 | > 90 days, < 1 yrs | R12 | 7 |
|  | 7 | > 90 days, < 1 yrs | R12 | 7 |
|  | 8 | > 90 days, < 1 yrs | R12 | 7 |
|  | 9 | > 90 days, < 1 yrs | R12 | 7 |
|  | 10 | > 90 days, < 1 yrs | R12 | 7 |
|  | 11 | > 90 days, < 1 yrs | R12 | 7 |
|  | 12 | > 90 days, < 1 yrs | R12 | 7 |
|  | 13 | > 90 days, < 1 yrs | R12 | 7 |
|  | 14 | > 90 days, < 1 yrs | R12 | 7 |
|  | 15 | > 1 yrs, < 2 yrs | R3 | 3 |
|  | 16 | > 1 yrs, < 2 yrs | R3 | 3 |
|  | 17 | > 1 yrs, < 2 yrs | R3 | 3 |
|  | 18 | > 1 yrs, < 2 yrs | R3 | 3 |
|  | 19 | > 1 yrs, < 2 yrs | R3 | 3 |
|  | 20 | > 1 yrs, < 2 yrs | R4 | 2 |
|  | 21 | > 1 yrs, < 2 yrs | R4 | 2 |
|  | 22 | > 1 yrs, < 2 yrs | R4 | 2 |
|  | 23 | > 1 yrs, < 2 yrs | R4 | 2 |
| Captive adults | 1 | > 3 yrs | C6 | 2 |
|  | 2 | > 3 yrs | C6 | 2 |
|  | 3 | > 3 yrs | C6 | 2 |
|  | 4 | > 3 yrs | C17 | 1 |
|  | 5 | > 3 yrs | C8 | 1 |
|  | 6 | > 3 yrs | C8 | 1 |
|  | 7 | > 3 yrs | C9 | 1 |
|  | 8 | > 3 yrs | C9 | 1 |
|  | 9 | > 3 yrs | C8 | 1 |
|  | 10 | > 3 yrs | C8 | 1 |
|  | 11 | > 3 yrs | C9 | 2 |
|  | 12 | > 3 yrs | C9 | 2 |
|  | 13 | > 3 yrs | C9 | 2 |
|  | 14 | > 3 yrs | C10 | 1 |
|  | 15 | > 3 yrs | C10 | 1 |
|  | 16 | > 3 yrs | C11 | 1 |
|  | 17 | > 3 yrs | C11 | 1 |
| Captive adolescents | 1 | > 90 days, < 1 yrs | C1 | 5 |
|  | 2 | > 90 days, < 1 yrs | C1 | 5 |
|  | 3 | > 90 days, < 1 yrs | C1 | 5 |
|  | 4 | > 90 days, < 1 yrs | C1 | 5 |
|  | 5 | > 90 days, < 1 yrs | C1 | 5 |
|  | 6 | > 90 days, < 1 yrs | C1 | 5 |
|  | 7 | > 90 days, < 1 yrs | C1 | 5 |
|  | 8 | > 90 days, < 1 yrs | C1 | 5 |
|  | 9 | > 90 days, < 1 yrs | C1 | 5 |
|  | 10 | > 90 days, < 1 yrs | C1 | 5 |
|  | 11 | > 1 yrs, < 2 yrs | C4 | 3 |
|  | 12 | > 1 yrs, < 2 yrs | C4 | 3 |
|  | 13 | > 1 yrs, < 2 yrs | C4 | 3 |
|  | 14 | > 1 yrs, < 2 yrs | C4 | 3 |
|  | 15 | > 1 yrs, < 2 yrs | C4 | 3 |
|  | 16 | > 1 yrs, < 2 yrs | C4 | 3 |
| Wild | 1 | Unknown | Wild | 11 |
|  | 2 | Unknown | Wild | 11 |
|  | 3 | Unknown | Wild | 11 |
|  | 4 | Unknown | Wild | 11 |
|  | 5 | Unknown | Wild | 11 |
|  | 6 | Unknown | Wild | 11 |
|  | 7 | Unknown | Wild | 11 |
|  | 8 | Unknown | Wild | 11 |
|  | 9 | Unknown | Wild | 11 |
|  | 10 | Unknown | Wild | 11 |
|  | 11 | Unknown | Wild | 11 |
|  | 12 | Unknown | Wild | 11 |
|  | 13 | Unknown | Wild | 11 |
|  | 14 | Unknown | Wild | 11 |
|  | 15 | Unknown | Wild | 11 |
|  | 16 | Unknown | Wild | 11 |
|  | 17 | Unknown | Wild | 11 |
|  | 18 | Unknown | Wild | 11 |
|  | 19 | Unknown | Wild | 11 |
|  | 20 | Unknown | Wild | 11 |
|  | 21 | Unknown | Wild | 11 |
|  | 22 | Unknown | Wild | 11 |

**Table S3. Annotation confidence score of opportunistic enteric zoonotic pathogenic OTUs.**

| Pathogen | OTU | Annotation confidence score |
| --- | --- | --- |
| *Campylobacter spp.* | B240 | 100% |
|  | B899 | 100% |
|  | B804 | 100% |
|  | B3 | 100% |
|  | B646 | 100% |
|  | B552 | 100% |
|  | B238 | 100% |
|  | B348 | 100% |
|  | B519 | 100% |
|  | B20 | 100% |
|  | B1253 | 100% |
|  | B652 | 100% |
|  | B1144 | 100% |
|  | B777 | 100% |
|  | B386 | 100% |
|  | B1116 | 100% |
|  | B1245 | 100% |
| *Mycoplasma spp.* | B36 | 67% |
|  | B101 | 100% |
| *Clostridium perfringens* | B559 | 100% |
| *Clostridium piliforme* | B29 | 67% |
|  | B1230 | 67% |
